# Influence of Drug-Carrier Polymers on Alpha-Synucleinopathies: A Neglected Aspect in New Therapies Development

**DOI:** 10.1155/2018/4518060

**Published:** 2018-02-21

**Authors:** C. Tonda-Turo, M. Herva, V. Chiono, G. Ciardelli, M. G. Spillantini

**Affiliations:** ^1^Department of Mechanical and Aerospace Engineering, Politecnico di Torino, Corso Duca degli Abruzzi 24, 10129 Turin, Italy; ^2^Department of Clinical Neurosciences, University of Cambridge, The Clifford Allbutt Building, Cambridge CB2 0AH, UK

## Abstract

Current therapeutic strategies to treat neurodegenerative diseases, such as alpha-synucleinopathies, aim at enhancing the amount of drug reaching the brain. Methods proposed, such as intranasal administration, should be able to bypass the blood brain barrier (BBB) and even when directly intracerebrally injected they could require a carrier to enhance local release of drugs. We have investigated the effect of a model synthetic hydrogel to be used as drug carrier on the amount of alpha-synuclein aggregates in cells in culture. The results indicated that alpha-synuclein aggregation was affected by the synthetic polymer, suggesting the need for testing the effect of any used material on the pathological process before its application as drug carrier.

## 1. Introduction

Neurodegenerative diseases, such as alpha-synucleinopathies, affect nerve cells function, leading to their death, disruption of neuronal network, and development of disease [1]. Alpha-synucleinopathies such as Parkinson's disease and Dementia with Lewy bodies are characterized by the presence of intracellular alpha-synuclein filamentous aggregates in degenerating cells in the form of Lewy bodies and Lewy neurites, while in multiple system atrophy they form glial cytoplasmic inclusions [1]. It is now clear that alpha-synuclein aggregates spread from one cell to the other, contributing to the progressive development of pathology.

Although intensively studied for many years, no treatment is currently available to stop progression of the neurodegenerative process. The approved pharmacological treatments available only provide symptomatic relief and a reduction in disease progression. One of the main factors affecting drug efficacy and utilization is the inability to reach the brain at a therapeutic concentration. To overcome this limitation, invasive treatments such as intracranial injection of hydrogels for drug delivery have been developed [2]. The use of a bioresorbable hydrogel loaded with drugs or engineered cells to achieve a direct drug delivery to the brain would ideally provide continuous drug supply at a clinically relevant concentration without the need of surgical intervention for its removal. Synthetic and natural polymers have been therefore investigated for intracerebral release of anti-inflammatory and neurotrophic factors leading to increased neuroprotection [2–4].

As an alternative to intracerebral delivery, the nasal route of administration has recently gained great interest for the treatment of neurodegenerative diseases, since it provides a noninvasive method for rapid drug adsorption bypassing the obstacle created by the blood brain barrier (BBB) [5]. The efficacy of the nasal drug uptake, compared to intraperitoneal route for example, has been demonstrated using different biomolecules, with the results showing that the amount of molecules reaching the brain is more than 5 times higher with the intranasal compared to intraperitoneal delivery [6]. Different formulations of polymers have been tested to maximize the drug uptake from the nasal cavity to the brain. Among them, mucoadhesive hydrogels have been shown to prolong the contact time between the drug and the nasal mucosa. The drug bioavailability is consequently increased, reducing the number of administrations needed to achieve the targeted therapeutic effect [7].

Synthetic, amphiphilic multiblock copolymers with thermosensitive behavior have been widely applied as injectable drug delivery systems [8–10]. Among them, Poloxamers, a family of triblock poly(ethylene glycol)-poly(propylene oxide)-poly(ethylene glycol) (PEG-PPO-PEG) hydrogels, have been extensively used [8]. Pluronic F-127 is one of the more widely applied Poloxamers in the biomedical field [11]. Chen et al. have recently developed a Pluronic F-127 based gel for the nasal administration of curcumin to the brain, demonstrating superior drug uptake compared to the intravenous administration route [12]. Strappe et al. have developed an injectable Pluronic F-127 gel for the intracranial delivery of a lentiviral vector to the brain [13].

However, despite being widely proposed and sometimes used, hydrogels for the intracerebral and nasal administration of drugs to the brain have not been adequately characterized as the presence of hydrogels and their dissolution products could interfere with the treated pathology.

In intracerebral application, hydrogels are in direct contact with the brain tissue and may interact with cells, causing unwanted side effects affecting cell behavior. In intranasal applications, the permeability of the nasal mucosa to hydrogel molecules has not yet been studied; this will be relevant in order to exclude the possibility that lower molecular weight molecules may cross the nasal barrier and reach the brain, with possible side effects.

Up to date, studies analyzing the influence of polymeric drug carriers on neurodegenerative pathologies have not been reported, and experimental methods for such an analysis are consequently missing.

In this work, we investigate the effect of PEG-PPO-PEG hydrogels on alpha-synuclein aggregation using an* in vitro* cellular model that was previously developed [14], where human neuroblastoma cells are infected with alpha-synuclein filamentous aggregates produced by protein misfolding cyclic amplification (PMCA) [14].

Results show that the presence of PEG-PPO-PEG increases the formation of alpha-synuclein fibrillary aggregates, which could induce unwanted contribution to pathology progression. These data support the need to investigate the effect of hydrogels in the pathological process before their use for drug delivery to the brain.

## 2. Materials and Methods

### 2.1. PEG-PPO-PEG Hydrogel Preparation

A PEG-PPO-PEG solution was prepared by dissolving 200 mg of Pluronic F-127 (Sigma-Aldrich) in 1 ml of Roswell Park Memorial Institute (RPMI) medium (Gibco) containing 10% fetal bovine serum (FBS, Invitrogen), 1% of penicillin/streptomycin (Gibco), and 1% L-Glutamine (Gibco) at 4°C and then adding 100 *μ*l of Pluronic 68 10% solution (Sigma Aldrich). The hydrogel was obtained by pouring 750 *μ*l of the final solution in a silicon mold which was left at 37°C for 30 minutes to achieve complete gelation [12]. Then, a volume of 1.5 ml RPMI medium was added. After 24 h, the supernatant containing PEG-PPO-PEG hydrogel dissolution products was collected and used for* in vitro* cell testing.

### 2.2. Cytotoxicity Assay: 3-(4,5-Dimethylthiazol-2-yl)-2,5-diphenyltetrazolium Bromide (MTT) Assay

MTT assay was performed to evaluate hydrogel cytotoxicity using human neuroblastoma cell line SH-SY5Y (ATCC® CRL. 2266™). SH-SY5Y cells were cultured on a 96-multiwell plate at a cell density of 8 × 10^4^ cells/well for 24 h to reach confluence. Then, the culture medium was removed and substituted with the supernatant collected from the PEG-PPO-PEG hydrogel (as prepared as described in par. 2.1) at 1 : 1 and 1 : 5 v : v dilution. Positive control (CTRL+) was obtained inducing complete cell death by adding 20% dimethyl sulfoxide (DMSO, Sigma-Aldrich) to medium, while normal medium was used for negative control (CTRL−). After 24 h incubation, the supernatant was carefully removed and the MTT assay was performed following supplier's instructions. Briefly, a volume of 100 *μ*l MTT solution (Sigma-Aldrich, 5 mg/ml in phosphate-buffered saline (PBS)) was added in each well and the cultures were incubated for 3 h. Then, MTT solution was removed and 100 *μ*l DMSO was added to each well and gently mixed to dissolve the formazan crystals. Absorbance was measured using an UV-Vis plate reader (Tecan Infinite M200PRO) at 570 nm wavelength. Cell viability was calculated as a percentage value compared to CTRL+. Six samples for each condition were used and experiments were performed three times. GraphPad Prism® software was used for one-way or two-way analysis of variance (ANOVA). ^*∗*^*p* < 0.05 was considered statistically significant.

### 2.3. Effect of PEG-PPO-PEG Hydrogel on Alpha-Synuclein Fibrils (*α*FF) Aggregation

SH-SY5Y cells were cultured on glass coverslip in a 24-multiwell plate at a cell density of 2 × 10^4^ cells/well for 24 h prior to infection. Then, fresh media containing 70 nM (a 1:1000 dilution from the 70 uM stock) of *α*FF, prepared following PMCA protocol developed by Herva et al. [14], were added and, at the same time, hydrogel supernatant at 1 : 5 v : v dilution was supplied to the infected cells. After 5 days, cells were fixed with a 4% paraformaldehyde solution (Sigma-Aldrich), permeabilized using 0.003% Triton (Sigma-Aldrich), and then stained using 4′,6-diamidino-2-phenylindole (DAPI, Sigma-Aldrich) 1 : 10000 diluted in PBS and pentameric form of formyl thiophene acetic acid (pFTAA kindly provided by Klingstedt et al. [15]) 1 : 10000 diluted in PBS for cell nuclei and *α*FF detection, respectively.

Cells were photographed under a fluorescence microscope (Leica CTR4000). Six images for sample were taken at 20x magnification and the experiment was repeated three times. Cell nuclei were counted using ImageJ to measure the number of adhered cells, while the amount of cells possessing *α*FF aggregates (*α*FF-cells) was quantified, counting the number of cells showing at least one green spot using ImageJ. Data were expressed as the average number of *α*FF cells with respect to the total number of adhered cells for each image ± standard deviation. Finally, the intensity of green spots within *α*FF cells was quantified using ImageJ processing tools.

## 3. Results

### 3.1. Cytotoxicity Assay: 3-(4,5-Dimethylthiazol-2-yl)-2,5-diphenyltetrazolium Bromide (MTT) Assay

**Figure 1:**
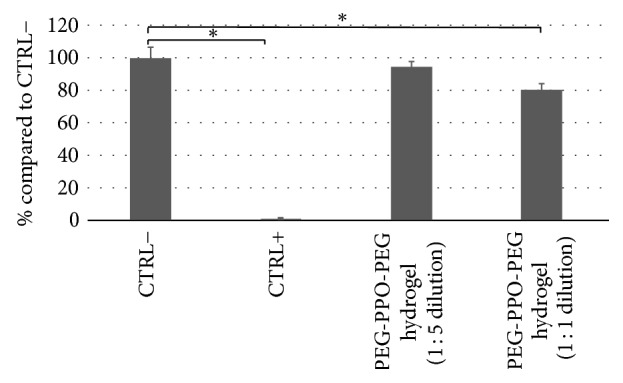
Viability of SH-SY5Y cells cultured in the presence of the extracts from PEG-PPO-PEG hydrogel at different dilution ratios (at 1 : 1 and 1 : 5 v : v dilution), compared to positive (culture medium) and negative (complete cell death induced by adding 20% of DMSO) controls. Asterisks denote statistically significant difference with ^*∗*^*p* ≤ 0.05 (ANOVA using GraphPad Prism® software).

To determine the toxicity of PEG-PPO-PEG, cells were exposed at two different dilutions (1 : 1 and 1 : 5 v : v dilution) of extracts from the hydrogels.  Figure 1 shows the viability of SH-SY5Y cells cultured in the presence of the extracts (dissolution products) from PEG-PPO-PEG hydrogels at different dilution ratios. Data were reported as percentage of negative control condition (CTRL−), where cells were cultured using normal medium. A positive control (CTRL+) was also obtained by adding 20% dimethyl sulfoxide (DMSO, Sigma-Aldrich) to medium to induce complete cell death. PEG-PPO-PEG dissolution products showed a good biocompatibility: only a slight reduction in cell viability (reaching 80% compared to CTRL−) was observed for nondiluted solutions (dilution 1 : 1), while no significant difference in cell viability was measured for 1 : 5 dilution (>90%) compared to control conditions (100%). The 1 : 5 dilution was therefore selected to test alpha-synuclein fibrils (*α*FF) aggregation in the presence of hydrogel dissolution products.

### 3.2. Effect of PEG-PPO-PEG Hydrogel on Alpha-Synuclein Fibrils (*α*FF) Aggregation

**Figure 2:**
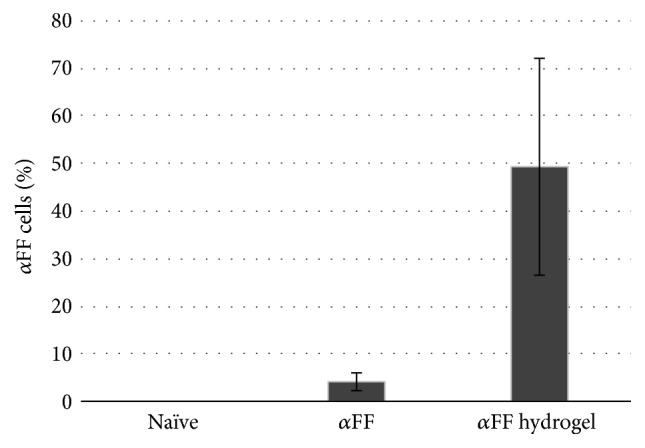
Histograms report the percentage of SH-SY5Y cells possessing *α*FF fibrils (*α*FF cells) with respect to SH-SY5Y cells adhered when SH-SY5Y cells were cultured in normal media (naïve), in media containing 70 nM of *α*FF (*α*FF), and in media containing both 70 nM of *α*FF and hydrogel supernatant at 1 : 5 dilution (*α*FF hydrogel).

**Figure 3:**
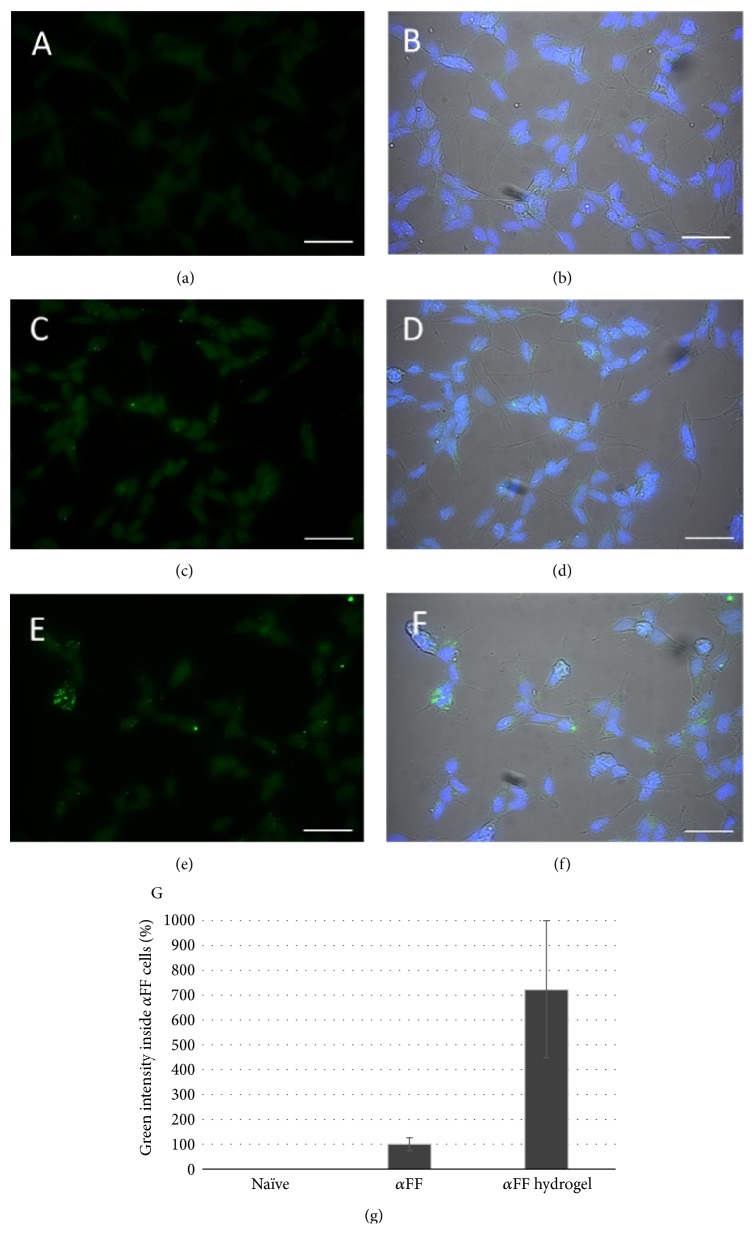
Fluorescence microscopy representative images of SH-SY5Y cells after 5 days of culture in normal media ((a) and (b)), in media containing 70 nM of *α*FF ((c) and (d)), and in media containing both 70 nM of *α*FF and hydrogel supernatant ((e) and (f)). Cell nuclei are reported in blue (DAPI staining) and *α*FF fibrils in green (PFTA staining). Bars: 50 *μ*m. (g) Green intensity inside *α*FF cells. Data are reported as percentage compared to green intensity inside *α*FF cells when SH-SY5Y cells were cultured with media containing *α*FF only.

The influence of hydrogel dissolution products on *α*FF accumulation by the SH-SY5Y cells was measured as a parameter to evaluate the interaction of drug carriers with pathology progression. Results showed that the presence of PEG-PPO-PEG dissolution products caused an increase in the amount of SH-SY5Y cells possessing alpha-synuclein fibrils aggregates (*α*FF cells) compared to the control (Figure 2). Then, each image was processed to quantify the green intensity ascribed to *α*FF fibrils within *α*FF cells. This analysis shows green intensity inside *α*FF cells from 4 to 10 times higher when *α*FF cells were cultured with media containing both *α*FF and PEG-PPO-PEG dissolution products compared to cells cultured in media containing *α*FF only (Figure 3), confirming the increase in the amount of *α*FF per cell when medium contains hydrogels dissolution products. Furthermore, when cells were cultured in the presence of both *α*FF and PEG-PPO-PEG dissolution products, cell clusters formed (Figures 3(e) and 3(f)), foreseeing an accelerated fibrils propagation within closer cells.

## 4. Conclusion

Drug carriers represent an effective tool to increase the ability of drugs to reach the target tissues. In the last years, the use of both intracerebrally injected and intranasally administered hydrogels for drug delivery to the brain has paved the way for the development of innovative therapies for the treatment of neurodegenerative diseases. When using intracerebrally administered hydrogels, cells are in direct contact with the carrier material and its dissolution products, while in the case of intranasal administration, only an amount of low molecular weight hydrogel dissolution products may pass through the nasal mucosa reaching the brain cells. However, in both cases, the effect of hydrogel dissolution products on the pathology progression requires investigation, particularly in diseases with protein aggregation like alpha-synucleinopathies, where it is known that protein aggregates spread from one cell to another. In this study, an* in vitro* method was used to analyze the influence of PEG-PPO-PEG hydrogel dissolution products on infection of SH-SY5Y cells with *α*FF. The experiments showed a relevant increase in the amount of SH-SY5Y cells possessing *α*FF fibrils (*α*FF cells) per image and an increase of *α*FF fibrils inside *α*FF cells, when cells were infected and cultured in the presence of hydrogel dissolution products. The ability of this hydrogel to enhance *α*FF aggregates could strongly affect the pathology progression, limiting the efficacy of the treatment inducing an unwanted contribution to aggregates formation. These findings highlight the importance of a more detailed characterization of the polymer carriers used for intracerebral or nasal drug delivery before exploring potential treatments for neurodegenerative diseases. Future work will need to be performed to determine* in vivo* whether the amount of hydrogel dissolution products that reach the brain is also promoting increased alpha-synuclein aggregation and whether there is a difference between intracerebral and intranasal injections. The development of smart polymers able to avoid unwanted effects that could affect the treatment outcomes is a key strategy to open the way for novel therapies based on drug delivery systems for neurodegenerative diseases.

## References

[1] Spillantini M. G., Goedert M. (2000). The *α*-synucleinopathies: Parkinson's disease, dementia with Lewy bodies, and multiple system atrophy. *Annals of the New York Academy of Sciences*.

[2] Pakulska M. M., Ballios B. G., Shoichet M. S. (2012). Injectable hydrogels for central nervous system therapy. *Biomedical Materials*.

[3] Nih L. R., Carmichael S. T., Segura T. (2016). Hydrogels for brain repair after stroke: an emerging treatment option. *Current Opinion in Biotechnology*.

[4] Khaing Z. Z., Thomas R. C., Geissler S. A., Schmidt C. E. (2014). Advanced biomaterials for repairing the nervous system: what can hydrogels do for the brain?. *Materials Today*.

[5] Hanson L. R., Frey W. H. (2008). Intranasal delivery bypasses the blood-brain barrier to target therapeutic agents to the central nervous system and treat neurodegenerative disease. *BMC Neuroscience*.

[6] B. Chauhan M., B. Chauhan N. (2015). Brain Uptake of Neurotherapeutics after Intranasal Delivery in Mice. *Journal of Neurology and Neurosurgery*.

[7] Chaturvedi M., Kumar M., Pathak K. (2011). A review on mucoadhesive polymer used in nasal drug delivery system. *Journal of Advanced Pharmaceutical Technology & Research*.

[8] Boffito M., Sirianni P., Di Rienzo A. M., Chiono V. (2015). Thermosensitive block copolymer hydrogels based on poly(E-caprolactone) and polyethylene glycol for biomedical applications: State of the art and future perspectives. *Journal of Biomedical Materials Research Part A*.

[9] Lee A. L. Z., Ng V. W. L., Gao S., Hedrick J. L., Yang Y. Y. (2015). Injectable biodegradable hydrogels from vitamin D-functionalized polycarbonates for the delivery of Avastin with enhanced therapeutic efficiency against metastatic colorectal cancer. *Biomacromolecules*.

[10] Cai B. L., Chang Y. G., Mei J. H. (2008). Thermoreversible gel-sol behavior of biodegradable PCL-PEG-PCL triblock copolymer in aqueous solutions. *Journal of Biomedical Materials Research Part B: Applied Biomaterials*.

[11] Al Khateb K., Ozhmukhametova E. K., Mussin M. N. (2016). In situ gelling systems based on Pluronic F127/Pluronic F68 formulations for ocular drug delivery. *International Journal of Pharmaceutics*.

[12] Chen X., Zhi F., Jia X. (2013). Enhanced brain targeting of curcumin by intranasal administration of a thermosensitive poloxamer hydrogel. *Journal of Pharmacy and Pharmacology*.

[13] Strappe P. M., Hampton D. W., Cachon-Gonzalez B., Fawcett J. W., Lever A. (2005). Delivery of a lentiviral vector in a Pluronic F127 gel to cells of the central nervous system. *European Journal of Pharmaceutics and Biopharmaceutics*.

[14] Herva M. E., Zibaee S., Fraser G., Barker R. A., Goedert M., Spillantini M. G. (2014). Anti-amyloid Compounds Inhibit *α*-Synuclein Aggregation Induced by Protein Misfolding Cyclic Amplification (PMCA). *The Journal of Biological Chemistry*.

[15] Klingstedt T., Shirani H., Åslund K. O. A. (2013). The structural basis for optimal performance of oligothiophene-based fluorescent amyloid ligands: Conformational flexibility is essential for spectral assignment of a diversity of protein aggregates. *Chemistry - A European Journal*.

